# War Experience, Daily Stressors and Mental Health Among the Inter-taliban Generation Young Adults in Northern Afghanistan: A Cross-Sectional School-Based Study

**DOI:** 10.3389/fpsyt.2022.877934

**Published:** 2022-05-17

**Authors:** Katayoon Razjouyan, Hossein Farokhi, Farah Qaderi, Pashtoon Qaderi, Seyed Javad Masoumi, Asghar Shah, Mohamad Amin Pourhoseingholi, Attaullah Ahmadi, Don Eliseo III Lucero-Prisno, Akihiko Ozaki, Yasuhiro Kotera, Jaffer Shah, Fawzia Negin, Shohra Qaderi

**Affiliations:** ^1^Department of Psychiatry, Imam Hossain Hospital, Shahid Beheshti University of Medical Sciences, Tehran, Iran; ^2^School of Medicine, Shahid Beheshti University of Medical Sciences, Tehran, Iran; ^3^Student Research Committee, School of Medicine, Shahid Behshti University of Medical Sciences, Tehran, Iran; ^4^Psychology and Educational Science Department, Balkh University, Balkh, Afghanistan; ^5^Department of Psychology, Faculty of Psychology and Education, University of Tehran, Tehran, Iran; ^6^Division of Biology and Medicine, Brown University, Providence, RI, United States; ^7^Department of Biostatistics, Shahid Beheshti University of Medical Sciences, Tehran, Iran; ^8^Medical Research Center, Kateb University, Kabul, Afghanistan; ^9^Department of Global Health and Development, London School of Hygiene and Tropical Medicine, London, United Kingdom; ^10^Jyoban Hospital of Tokiwa Foundation, Iwaki, Japan; ^11^School of Health Sciences, University of Nottingham, Nottingham, United Kingdom; ^12^Drexel University College of Medicine, Philadelphia, PA, United States; ^13^Afghanistan National Charity Organization for Special Diseases, Kabul, Afghanistan; ^14^Faculty of Medicine, Balkh University, Balkh, Afghanistan

**Keywords:** mental health, young adults, war experience, daily stressors, Taliban fall, post-Taliban generation, conflict zone, Afghanistan

## Abstract

**Objectives:**

The specific objectives of the study are to examine the mental health (depression and anxiety) of the first generation of post-Taliban government and compare these measures with its preceding generation, and to assess war experience of the first generation of post-Taliban government. We also wanted to assess the daily stressors and their contribution to the mental health, and to assess mental health as a result of war experiences and daily stressors with respect to demographic measures such as sex, marital status, age, mother's age, birth order, and ethnicities.

**Methods:**

In a cross-sectional design, 621 high school students, were randomly selected to participate in the study to assess war experience, daily stressors, and mental health among the first generation of young adults under post-Taliban government.

**Results:**

The participants had 17.37 ± 0.9 mean years of ages, 94.8% of them were unmarried. Poor mental health was significantly associated with higher exposure to war, but not with the age of participants (*P* = 0.08). There was no association between war experiences and the age and ethnicity of our participants (*p* = 0.9, *p* = 0.7). Age differences were negligible for daily stressors too (*P* = 0.07). Daily stressors scores were higher for female than male students (*P* = 0.02). The majority of young adults surveyed, declared themselves in agreement with statements such as the security situation in Afghanistan makes me frustrated (56%), air pollution as a concern (41%), and not having anyone to talk about what is in their heart (28.8%). Gender differences were highly significant for mental health, as appraised by both The Hopkins Symptoms Checklist (HSCL) –depression and HSCL-anxiety. Girls presented higher rates of depression, anxiety, and daily stressors than boys, and boys presented higher rates of war experiences than girls.

**Conclusion:**

War experience, daily stressors, and mental health were irrelevant with age, ethnicity and marital status. Factor such as being the first-born child of the family, higher reported war experiences, and daily stressors all negatively impact mental health. Alongside war and its direct effects, the existing socio-cultural context must be considered as a potential factor mediating the mental health of girls in Afghanistan.

## Introduction

Mental health is an integral and essential component of children's and adolescent's healthy development, but has been treated with low priority in Afghanistan (1). Despite 17 years having lapsed since the fall of the Taliban in Mazar-e-Sharif by the Afghan Northern Alliance in December 2001, the intervention of the international community has not led to an overall liberation of the Afghans. Afghanistan remains a conflict zone with much of the country controlled by warlords and terrorist attacks continuing in various regions of the country (2–5). With poor living conditions due to broken economy and poor governance, low life expectancy (61/64), high maternal and infant mortality rate, low level of literacy, widespread inequalities between gender and social economic and drug-based economy (60% of gross domestic product), Afghanistan is ranked near the bottom of the World's Human Development Index (168 out of 188 countries) (3–9).

According to the World Health Organization (WHO), youth exposed to war and home displacement may develop mental disorders including depression, suicide ideation, Post-Traumatic Stress Disorders (PTSD), and substance abuse (5). Armed violence and war in low- and middle-income countries (LMICs) pose a greater burden on existing mental health infrastructure due to a lack of resources (10). Song et al. highlight the need for more research to better characterize the mental health needs of people in LMICs. Our investigation aims to contribute to this call for research in the Afghanistan context. Researchers have found that the extent of war exposures is significantly associated with PTSD, in a conflict setting (11). Moreover, they conclude that the association between exposure to armed conflict and resulting psychological stress was significantly mediated by daily stressors for post-traumatic stress manifestations (11). Organized war and armed conflict produce and heighten daily stressors including poverty, social alienation, access to housing, and familial structure (12). Researchers have delineated a psychosocial model that includes both exposure to war and daily stressors as mediators of mental health (12). The inclusion of daily stressors is critical because exposure to the violent conflict can generate daily stressors and so these daily stressors play a role in the relationship between exposure to war and subsequent psychological distress (12). It is the continuing impact of political violence on people's lives that contributes to psychological distress (13). The researchers here distinguish between daily stressors that are potential traumatic events (PTEs) and other stressors. It is postulated that such PTEs contribute to chronic activation of the stress system and consequently poorer mental health over time (13).

As of today, every child born and raised in Afghanistan has experienced war and conflict in the country (14). Approximately 17% of the adult population in Afghanistan suffers from mental health disorders (15). Literature on mental health conditions in Afghanistan represent a high prevalence of depression, anxiety and post-traumatic disorders (3, 16, 17). According to a 2015 study, young adults in Kabul present with poor mental health status (18). These conditions have contributed to direct traumatic exposures as direct impact of armed conflicts on top of poor living standards, bad governance, and insecure family environment which have significant effect on poor mental health (17, 19). Young adults are a significant agent for change and economic power of the future of Afghanistan but become victims of these situations. Many children are pressured to work before the age limit of 18 due to low socio-economic status (20). This is contrary to Afghanistan's Labor Law, which stipulates that the minimum age for employment is 18 and children aged 14 and younger are not allowed to work. Partial security after Taliban's fall, led to an exponential increase in school attendance for both girls and boys in the country, specifically in Mazar-e-Sharif (the capital of Balkh province) (21, 22). We decided to use schools as potential sites for intervention to address young adult's mental health. Prior to the implementation of interventions, we wanted to understand the state of mental health by drawing community samples about the war experiences and daily life stress in the first generation of young adults under post-Taliban government in Mazar-e-Sharif. The specific objectives of the study are to examine the mental health (depression and anxiety) of the first generation of post-Taliban government and compare these measures with its preceding generation, and to assess war experience of the first generation of post-Taliban government. We also wanted to assess the daily stressors and their contribution to the mental health, and to assess mental health as a result of war experiences and daily stressors with respect to demographic measures such as sex, marital status, age, mother's age, birth order, and ethnicity.

## Methods

### Survey Design

**Figure 1:**
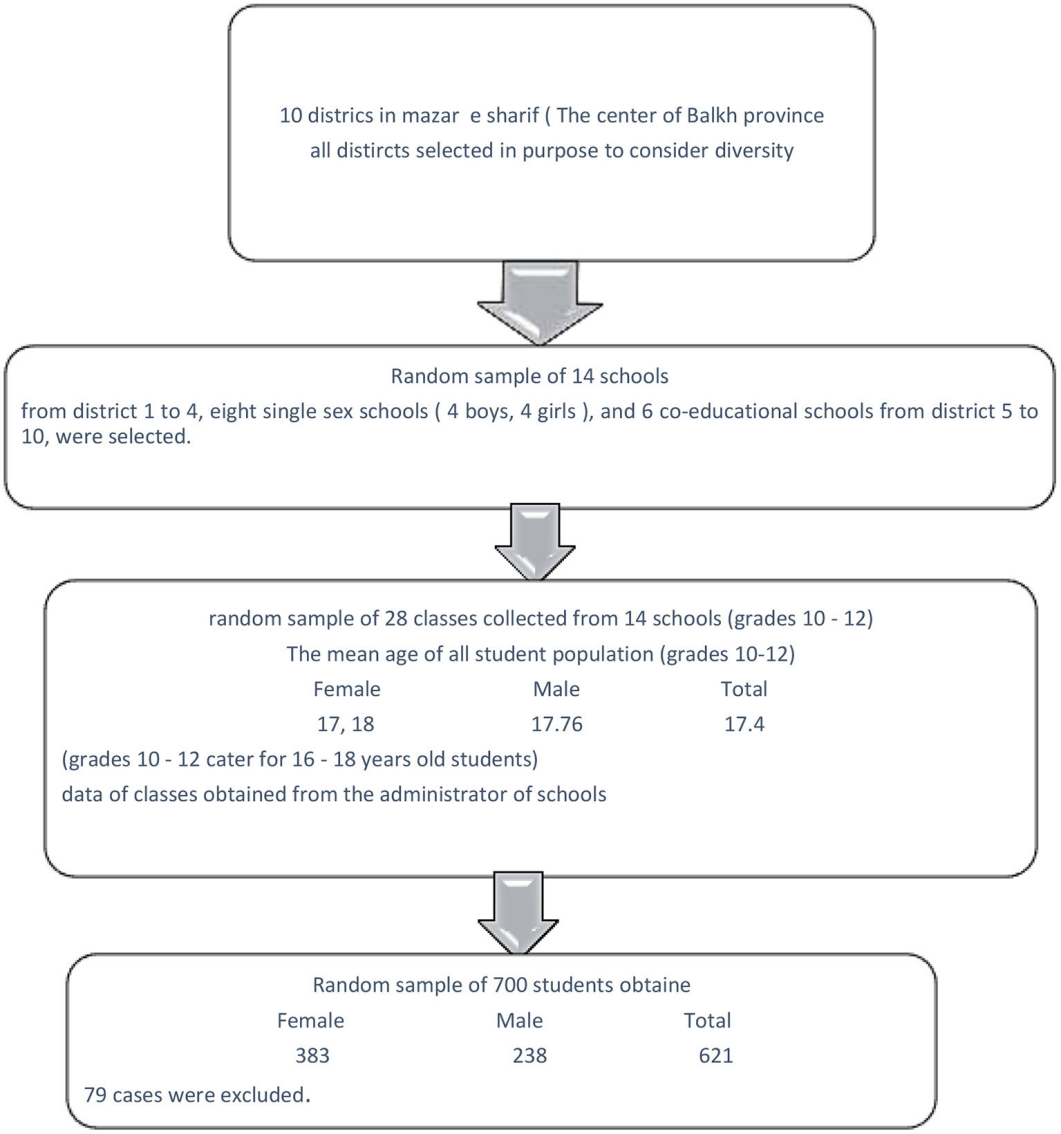
Sampling for a three stage, stratified random survey.

From the 20th of January to March 30th, 2018, we employed a cross-sectional design, wherein participants were included randomly. Participants were high school students aged between 16 and 18 years old from Mazar-e-sharif. This is the center of Balkh province which is known to have one of the high attendance in school in Afghanistan. Mazar-e-Sharif province has 10 local districts. Three-stage school-based sampling was used in this survey (Figure 1). The first stage of sampling considered all districts having a wide range of social and economic experiences. To calculate sample size, we used the limit of statistical significance α = 0.05 with 95% confidence intervals, and an estimated design effect of 2.5. Across these assumptions, 700 cases were deemed as adequate for estimation of the prevalence of the mental health status of the population. For the second stage of sampling, we randomly selected 14 schools which have high attendance in school. These are from districts 1–4. Eight segregated schools (four for boys and four for girls), and six co-educational schools from districts 5–10, were selected.

For the third stage of sampling, a random sample of 28 classes was collected from grades 10–12 with a minimum of 50 participants from each school. At the beginning of our survey, we randomly selected students from the class attendance list, though all the students were willing to participate in the study. We surveyed the entire members of one or two classes. Before the distribution of questionnaires, we obtained oral consent from all the students. Almost all students agreed to take part in the study and answer the questionnaires because of the novelty of our research. Due to the literacy level of the participants, we were able to maintain the same environmental conditions thus preventing response biases in our study. Afghans are not normally comfortable to talk about their distressing emotions. In this study, everyone got their own questionnaire and completed answering by themselves without an interviewer reading it aloud or them answering it for the interviewer in an audible voice. Most of the participants were asked whether their participation would benefit them with regards to their mental health or not. They were told that their responses will be gathered for research purposes only and findings will be shared back to the school (Figure 1).

The survey team had three staff that included two medical students and a driver. The two medical students were educated in Mazar-e-Sharif and were known by the students thus facilitating easy rapport with the students. The team moved sequentially from school to school which maximized efficiency in data collection and quality. The questionnaires were filled by students for ~15 min to complete. The protocol was approved by Shahid Beheshti University of Medical Science, Balkh education presidency and by all subsidiary school directors. As such, all ethical considerations were in order throughout the length of this study. One exclusion criteria for the study was not being part of the first generation young adults under the post-Taliban government.

### Screening Instruments

We used screening instruments for measuring mental health, which were chosen based on traumatic events considered, culturally specific attitudes and relevant psychometric properties for the war- affected population. Our study comprises three standard instruments: the Afghan Stressors Scale (ADSS), Afghan War Experiences Scale (AWES), and The Hopkins Symptom Checklist-25 (HSCL) (3, 17).

Independent variables considered are:

1. Socio-demographic characteristics such as age (the age of respondents, their fathers and mothers), gender, marital status, ethnicity, past educational gap, birth order, past exam failed, and house location.
2. War experiences such as exposure to war-related violence and loss was measured by the Afghan War Experiences Scale (AWES). This scale contains 17 war-related items; responses are rated on a 3-point Likert scale, (1) Never, (2) once, and (3) more than once. The sum of scores, yielding within a range of 17–51, with excellent reliability across gender (Cronbach‘s alpha = 0.94 for women and 0.91 for men) (23).
3. Daily stressors measured by the Afghan Daily Stressors Scale (ADSS). The ADSS was developed by Professor Miller with Dr. Omidian and other Afghani colleagues, using a multi-step process in Kabul. This culturally-grounded questionnaire contains 26 items which ask respondents to indicate how stressful they have found each item during the past month, the responses are rated on a 3-point Likert scale, (1) not stressful, (2) somewhat stressful, and (3) very stressful. The sum of scores, yielding a total score of 26–79. The ADSS is useful in assessing a wide range of daily stressors such as financial hardships, health problems, social isolation, family conflicts and environmental concerns. The reliability of ADSS varied by gender (Cranach's alpha = 0.7 for women and 0.58 for men). ADSS was also validated as a screening tool in a community setting in Pakistan (18).

Dependent variables considered are:

Mental health which measured depression and anxiety using the Hopkins Symptom Checklist (HSCL-25) (21). The HSCL contains 25-items that includes 15 items of depression and a 10-item subscale for anxiety with each item scored from 1 to 4 (24), 1 (“not at all”), and 4 (“extremely”). The HSCL has been widely used in studies on refugees and other war-affected populations (3, 23), and has demonstrated good reliability and validity.

### Statistical Analysis

All statistical analyses were conducted using SPSS version 16 for Windows. All numerical data were expressed as mean ± SD and percentage values for analysis, responses to Likert scale questionnaires were summed across constituent items, to yield total scores for each participant. Both categorical and continuous variables were subjected to bivariate test to examine association with ASCL scores using one-way ANOVAs, independent sample *t*-test, and Pearson‘s correlations were applied where appropriate. All the data were screened for linearity and normality with *p* < 0.05 considered as statistically significant for this study. The major number of analyses were conducted separately for each gender.

## Results

A total of 621 participants with 17.37 ± 0.9 mean years of age, completed the three questionnaires. Their father's and mother's mean of ages were 50.12 and 43, respectively. 62.9% (excluding 40 males who met the exclusion criteria) of our participants were females and 37.1% males, most were unmarried (94.8%). Nine percent of our participants had past educational deprivation and 29% reported failure in some school exams. The participants were predominantly Tajik (68.1%), Hazara (12.5%) and Pashtun (10.2%), which reflect the predominance of Tajik in Mazar-e-Sharif. Almost all (99.9%) participants had both parents still alive. Most of the students (67%) were the fourth eldest descendants (according to the birth-order) of their families (Table 1).

**Table 1:** Descriptive statistics: Independent variables.

**Characteristics**	**Female = 363 (100%)**	**Male = 214 (100%)**
**Age**		
**Participants**		
≤ 15 years	6 (1.7%)	0 (0%)
16–18	336 (92.6%)	184 (86%)
≥19	21 (5.8%)	29 (13.6%)
Parents age	Mothers	Fathers
≤ 39	179 (30%)	35 (6%)
40–59	357 (62%)	422 (73%)
≥60	16 (2.8%)	94 (16%)
**Ethnicity**		
Tajik	253 (69.7%)	115 (53.7%)
Hazara	13 (3.6%)	20 (9.3%)
Pashtun	33 (9%)	26 (12%)
Ozbek	34 (9.4%)	38 (17.8%)
Turkman	1 (0.3%)	8 (3.7%)
**Marital status**		
Single	341 (93.9%)	206 (96.3%)
Married	22 (6%)	8 (3.7%)
**Order of birth**		
≤ 4	235 (64.7%)	151 (70.6%)
5–7	99 (27.3%)	41 (19.2%)
≥8	23 (6.3%)	16 (7.5%)
**Past educational deprivation**		
Yes	30 (8.3%)	22 (10.3%)
No	328 (90.4%)	192 (89%)

In our study, the mean score of HSCL–depression and HSCL-anxiety were 28.45 ± 10 and 19.39 ± 7, respectively, to examine the mental health (depression and anxiety) of the first generation under the post-Taliban government and to compare these measures with its preceding generation. Positive correlation were obtained between HSCL-Depression and HSCL-anxiety (*P* < 0.001). For assessing war experience of the first generation of post-Taliban government, the AWE-17 scores ranged from 17 to 51 with an average score of 19.32 ± 3.72. The mean score of ADSS-26 was 42 ± 10 (Table 2). The majority of participants declared themselves in agreement with statements such as the security situation in Afghanistan makes me frustrated (56%), air pollution as a concern (41%), and not having anyone to talk about what is in their heart (28.8%), (Table 3). Five percent of our participants suffered from the loss of family income frequently, 21 and 18.5% of cases suffered from not being able to buy the things their family needed and loss of their income, respectively (Table 4).

**Table 2:** The relation between mental health, war experiences and daily stressors with age and gender.

**Questionnaire**	**Mean ± SD**	**Age** ***P*-value**	**Gender differences** ***P*-value**	**ADS** ***P*-value**	**HSCL-A** ***P*-value**	**HSCL-D** ***P*-value**	**AWE** ***P*-value**
ADSS	42 ± 10	0.5	0.02		<0.001	<0.001	<0.001
**HSCL-25**							
HSCL-A	19.39 ± 7	0.6	<0.001	<0.001			<0.001
HSCL-D	28.45 ± 10	0.9	<0.001	<0.001			<0.001
AWE	19.32 ± 3.72	0.3	0.01	<0.001	<0.001	<0.001	

*ADSS, Afghan Daily Stressors Scales; HSCL, Hopkins Symptom Checklist (HSCL-25), HSCL- anxiety, HSCL-depression; AWE, Afghan war expenses*.

**Table 3:** Descriptive statistics of Afghan daily stressors scales^*^ with three likert.

**During the past month, how stressful has each of the following been for you?**	**Not at all stressful (%)**	**Somewhat stressful (%)**	**Very stressful (%)**	**Gender differences** ***P*-value^**α**^**
Not having enough money to pay for things my family needs.	44.9	38.6	16.5	–
Not feeling safe in my home.	62.6	30.2	7.3	–
Not feeling healthy.	48.2	40.6	11.3	–
Not being able to find work.	50	30	20.8	0.003
Not feeling safe walking around outside of my home.	52.9	29.5	17.5	–
Not feeling happy with my family.	63.4	29	7.5	0.03
Not being able to afford medicines I need.	66.7	23.9	9.4	–
Problems with my teeth.	62.7	27.9	9.4	0.03
My family member being sick.	39	36.9	24	0.003
Feeling lonely.	51.5	29.5	19	–
Feeling like people do not give me enough support.	55	27.9	17	0.01
Being beaten by a family member.	81.6	12.7	5.7	–
Financial problems.	42.8	37	20	–
Overcrowding in my house.	65.9	26.9	7.3	–
The physical condition of my house.	68.6	25.3	6	–
Conflict in my home.	64.3	25.5	10	–
Missing relatives who live far away.	40.7	38	21	–
The security situation in Afghanistan.	16	27.7	56	–
Not having anyone I can really talk to about what is in my heart.	41	30	28.8	–
Air pollution.	20	38	41.8	0.007
Roadblocks.	36.6	40	23.6	–
Not having children of my own.	79	11	10	–
Having too many children in the house.	72.3	18.5	9.2	–
Being unable to provide for my children's needs.	72	18	10	–
Being unable to read or write.	67.5	14	18.5	–
Not owning my own home.	65.5	15.3	19.2	–

* *Afghan daily Stressors, were used to develop a culturally-grounded instrument reflecting a range of stressful social conditions (17)*.

α *P-value for gender differences on responses to the 3-point Likert-scale*.

**Table 4:** Descriptive statistics of Afghan War experiences scale.

AWE^*^= 27	**No = *N* (%)**	**Once = *N* (%)**	**Several = *N* (%)**
Destruction of your house	526 (91.2)	43 (7.5)	8 (1.4)
Destruction of your village or neighborhood	495 (85.8)	62 (10.7)	20 (3.5)
Participation of one or more family	504 (87.3)	59 (10.2)	14 (2.4)
Rocket landing on your house	547 (94.8)	26 (4.5)	4 (0.7)
Death of a family member	501 (86.8)	67 (11.6)	9 (1.6)
Injury of a family member	497 (86.1)	72 (12.5)	8 (1.4)
Loss of your family income	443 (76.8)	107 (18.5)	27 (4.7)
Disappearance of a family member	534 (92.5)	34 (5.9)	9 (1.6)
Separation from a family member	509 (88.2)	58 (10)	10 (1.7)
Becoming a refugee from your	465 (80.6)	97 (16.8)	4 (0.7)
Being injured yourself during the war	557 (96.5)	16 (2.8)	4 (0.7)
Losing your property and wealth	486 (84.2)	75 (13)	14 (2.4)
Being put in jail	552 (95.7)	20 (3.5)	5 (0.9)
Being beaten	529 (91.7)	40 (6.9)	8 (1.4)
Having a family member in jail	526 (91.2)	46 (8)	5 (0.9)
A family member being beaten	519 (89.9)	54 (9.4)	4 (0.7)
Not being able to afford to buy the thing your family needed	454 (78.7)	123 (21.3)	0

* *Afghan war experiences*.

Gender differences were highly significant for differences in reported mental health, as appraised by both HSCL –depression and HSCL-anxiety. In our study, HSCL –depression and anxiety scores were higher for females than males (*p* < 0.001). Most of our participants had no experience of being injured, jailed or tortured during war. However, poor mental health status was significantly associated with higher exposure to war experience irrespective of gender and marital status (*P* < 0.001). High level of war experiences was significant among men (*P* = 0.01), with mean scores of 19.83 for males and 19.01 for females. Poor mental health was significantly associated with higher exposure to war, but not with the age of participants (*P* = 0.08). There was no association between war experiences and the age and ethnicity of our participants (*p* = 0.9, *p* = 0.7). Age differences were negligible for daily stressors too (*P* = 0.07).

Daily stressors scores were higher for female than male students (*P* = 0.02), and six items were significantly more stressful for females than males in this study, including “Not being able to find work,” “Not feeling happy with my family,” “Problems with my teeth,” “My family member being sick,” “Air pollution,” “Feeling like people don't give me enough support.” Furthermore, higher HSCL scores were significantly associated with high daily stressors (*P* = 0.01). Headache, feeling fearful, crying easily and feeling lonely among girl students and heart pounding or racing and loss of sexual interest or pleasure among boys' students were chosen extremely on four Likert scales. Compared to the first four family members, last family members experienced higher daily stressors (*P* = 0.04) (Table 4). Also, negative relation was found between having a younger mother (age < 39) and higher daily stressors scores (*P* = 0.04).

## Discussion

Despite the fall of the Taliban, every child born and raised in Afghanistan has experienced war and conflict (2, 14, 15). Our school-based study was conudcted in Mazar-e-Sharif, where school attendance is high, affording a climate of improved security (14). This study yielded systematic data regarding the investigation of mental health, war experiences and daily stressors among the first generation of young adults after the overthrow of the Taliban government.

On a design level, our study shows that schools are a valuable source of ascertaining mental health and mental health related factors among school-age young adults due to their feasibility, high national-diversity (Tajki, Hazara, Pashtoon, Ozbek and Turkman) and the student's willingness and pleasure to answer questions about their feelings and emotions. Our participants reported that they had never been asked about their feelings, emotional status and daily stressors, as has been reported in previous studies (25, 26). This finding raises the awareness of the importance of providing an opportunity for students to talk about their feelings and emotions, while underscoring a school-based intervention.

We found that daily stressors are stronger predictors of mental health than war experiences. These stressors were reported significantly higher in girls than in boys, in congruence with the study by Miller et al. (17), but not with Panter-Brick et al. (26). This result emphasizes the importance of community structure and cultural barriers for Afghan girls that must exist within broader sociocultural and political frameworks. Negative relation was found between having a younger mother (age < 39) and higher daily stressors scores, in congruence with the findings by Tearne et al. (27).

Our work contributes to the broader corpus of literature on youth mental health and resilience amid conflict and war in Afghanistan. Afghanistan's conflict setting is both rural and urban in nature, with an estimated 9.4 million people in need of humanitarian assistance (28). Moreover, in most provinces there is a lack of comprehensive emergency obstetric and newborn care (CEmONC). Also, economic accessibility to care is curbed by the introduction of now common user fees at both public and private health centers at the tertiary level in Afghanistan (28). Heinous attacks of this nature are a reminder of the continued violence experienced by the citizenry and the critical need to address the mental wellbeing of the population specifically in the Afghanistan context. A previous analysis of mental distress in Afghanistan found that vulnerable populations, including minority ethnic groups, the widowed, the elderly, etc., were at a heightened risk of experiencing mild to severe mental health problems (29). Trani et al. conclude that mental health is a function of a dynamic array of sociodemographic characteristics, economic and cultural contexts.

The first systematic, longitudinal study of measures of mental health and resilience in Afghanistan found that poor mental health was driven predominantly by gender, trauma exposure, caregiver wellbeing, and geographical area (30). The researchers go on to attribute these factors to Afghan girls being more than two times likely to have measures indicative of psychiatric disorder compared to boys. They also determined that “everyday violence” including familial or community level acts of violence and exposure to deleterious social and economic stressors, the effect of which effected the home environment, to be of equal importance to “militarized violence” (30).

In turn, we position the present work as building off this base by investigating the daily stressors that are present in the lives of young adults attending the schools of Mazar-e-Sharif. Previous literature also points to the need for public health researchers and scientists not to focus solely on the macrolevel effects of war and conflict. This is because a focus on the macrolevel alone leads to the erasure of the direct daily lived experiences of the individuals afflicted with the effects of the conflict. Ventevogel et al. (31) articulate this point by recommending researchers to investigate the primary factors mediating mental health, while accounting for violence at the family level, trauma, structural barriers, etc. They see this is improved understanding as a way to better informing potential psychiatric interventions of children and adolescents in conflict-afflicted contexts.

Moreover, research on resilience in humanitarian contexts has emphasized the use of multilayered psychosocial care that addresses health, familial status and dynamics, economic situation, and other layered factors contributing to one's mental health (32). This is most clearly seen in our inclusion of salient items measured through the Afghan Daily Stressors Scale (ADSS). On the factor of familial status and dynamics, prior research deemed the family as being the only stable institution in Afghanistan for socioeconomic development at the individual level (33). Moreover, this stability translates into youth mental health as domestic violence and inadequate caregiver mental health prove detrimental to resilience, even over the effects of direct war-violence (33). Thus, social capital and the broader contexts governing mental health are important to consider for public health researchers to understand how to best address the status of mental health among the youth in Afghanistan after the fall of the Taliban government in Mazar-e-Sharif. Finally, we position this literature on the global scale as efforts to understand and apply global mental health increase in prevalence (34). Specifically, by understanding better the regional-specific mental health mediators in Mazar-e-Sharif, the lessons may be transferred and applied in other contexts, where policy positions may be better informed by information such as salient mediators of resilience, effective psychometric scales, and other predictors of mental health.

Overall, the relation between war experiences and daily stressors was significant, the higher the exposure to war, the higher the daily stressors score obtained. Generally, the mean of the daily stressors in our study was 10 scores less than ADSS cut off points. For girls, items such as unable to find work, not feeling happy with family, teeth troubles, sickness of the family member and air pollution were significantly stressful. We found that there is a strong relation between mental health and daily stressors and this relation was stronger among females than males (*p* < 0.05), this result is the same as that found by Miller et al. (17). This result supports that not only war, but other invisible wounds cause deep scars on the mental health of girls such as social restriction, cultural barriers, low level of family and community support for girls, religious restrictions and households factors.

The majority of our participants declared themselves in agreement with statements such as dissatisfaction with the security situation of Afghanistan, air pollution as a concern, and not having someone to talk about what is in their heart. Despite the higher level of daily stressors among girls, war experiences were significantly higher among boys, as shown in a previous study (17). This is due to socio-cultural contexts in which men are the main breadwinner and the frontline in any social conflict. On the other hand, within the Afghanistan context, women are routinely encouraged to stay at home and observe housekeeping roles.

Approximately 99% of our participants have parents who are still alive, slightly dissimilar to one study (6) where one in ten children have lost one or both of their parents. High prevalence of mental health problems associated with higher war experiences, which underscores the direct effect of war on mental health, nevertheless ethnicity and age did not affect war experiences in this study, however, it takes issue with the previous study that ethnicity was recognized as an influential factor on mental health, which may be attributed to the dominancy of the Tajik ethnicity in Mazar-e-Sharif (6, 18).

The mean score of war experiences obtained 12 scores lesser than the AWES mean showing a dramatic decrease in war experiences; and the majority of our participants had no experience of bad injury, imprisonment, or torture (*p* < 0.05). The mean participant score for HSCL-A and HSCL-D obtained was 19.39 and 28.45 and mean Miller's study scores were 27.66 and 36.76 for HSCL-A and HSCL-D, respectively. This shows a significantly lower prevalence of mental health problems among young adult school goers in Mazar-e-Sharif. These findings are in contrast to older studies that observed a higher prevalence of mental problems among Afghans (3, 6, 17, 18). In agreement with previous studies, poor mental health was significantly higher among females, both in measures of anxiety and depression, however ethnicity, age (age of participants, mother's age and father's age), and marital status did not significantly affect mental health status. To be the first four-member of the family (birth order), negatively affected mental health (35).

In the HSCL questionnaire, headache, feeling fearful, crying easily and feeling lonely among girl students and heart pounding or racing and loss of sexual interest or pleasure between boy students were chosen extremely, on four Likert scale. According to the most recent survey of women between 20 and 24 years old, 34.8% of them were married by age of 18 (27, 36), meanwhile in Alemi's study 23.36 was the mean age in Kabul, wherein 38.7% of participants were married. However, in our study, over 94% of males and 96.3% of females were unmarried. Finally, more than 90% of our cases did not have past educational deprivation.

## Conclusion

Our finding suggests that adolescents in Mazar-e-Sharif present good mental health status, low war experiences and low level of daily stressors than previous studies have shown. However, in this present study females reported higher rates of depression, anxiety and daily stressors than boys, and boys presented higher rates of war experiences than girls. All three items were not associated significantly with age, ethnicity and marital status. Being the first child of the family, higher reported war experiences, and daily stressors, negatively impact mental health. Past educational deprivation and underage marriage were seen at low frequencies. Alongside the direct implications of war, existing socio-cultural contexts must be considered as a potential factor influencing the mental health of girls in Afghanistan.

## Limitations

We observed a number of limitations in the study. First, we failed to clarify a relative contribution of war experience and daily stressor on mental health of the considered students. This limited an implication of this study to develop potential countermeasures for the students' mental health problems. Second, although, both questionnaires of AWES and ADSS have high consistency in our study, they were created in a Kabul setting which may have some differences to the Mazar-e-Sharif context in this study. Some of the psychometric questionnaire items may not apply to Mazar-e-Sharif. For example, traffic has never been a significant stressor for Mazar-e-Sharif residents. Moreover, this study was carried out by school attendees, the mental health state of non-young school goers remains unknown. Future public health researchers may find it fruitful to expand on these findings by broadening the study sample to include young-school aged children. Among these students, we also do not know the existing mental health status, or if student participants were on medication for psychiatric disorders. We did not obtain information on socio-economic status of the participants. However, a large percentage of Afghans including Mazar-Sharif have low socio-economic status.

## Suggestion

We have found schools as a viable source of collecting information and potential point of intervention. There is no existing school-based mental health intervention in Mazar-e-Sharif. It could be a novel and helpful mode of therapy for all students.

## Data Availability Statement

The raw data supporting the conclusions of this article will be made available by the authors, without undue reservation.

## Author Contributions

KR, FQ, PQ, SQ, and HF conceived and designed the study and wrote the manuscript. JS, AA, and FQ helped collect data. MP, AS, and SM performed the statistical analysis and wrote the manuscript. DL-P and YK confirmed the eligibility of the participants' for the study. SQ, FN, and AO supervised the whole study and approved the final version of the manuscript. All authors contributed to the article and approved the submitted version.

## Conflict of Interest

The authors declare that the research was conducted in the absence of any commercial or financial relationships that could be construed as a potential conflict of interest.

## Publisher's Note

All claims expressed in this article are solely those of the authors and do not necessarily represent those of their affiliated organizations, or those of the publisher, the editors and the reviewers. Any product that may be evaluated in this article, or claim that may be made by its manufacturer, is not guaranteed or endorsed by the publisher.
